# Capillary electrophoresis mass spectrometry identifies new isomers of inositol pyrophosphates in mammalian tissues†

**DOI:** 10.1039/d2sc05147h

**Published:** 2022-12-05

**Authors:** Danye Qiu, Chunfang Gu, Guizhen Liu, Kevin Ritter, Verena B. Eisenbeis, Tamara Bittner, Artiom Gruzdev, Lea Seidel, Bertram Bengsch, Stephen B. Shears, Henning J. Jessen

**Affiliations:** a Institute of Organic Chemistry, Faculty of Chemistry and Pharmacy, University of Freiburg 79104 Freiburg Germany; b CIBSS – Centre for Integrative Biological Signaling Studies, University of Freiburg Germany Henning.jessen@oc.uni-freiburg.de; c Signal Transduction Laboratory, National Institute of Environmental Health Sciences, National Institutes of Health Research Triangle Park NC 27709 USA shears@niehs.nih.gov; d Clinic for Internal Medicine II (Gastroenterology, Hepatology, Endocrinology and Infectious Diseases), Freiburg University Medical Center, Faculty of Medicine, University of Freiburg Freiburg Germany; e SGBM – Spemann Graduate School of Biology and Medicine, University of Freiburg Germany

## Abstract

Technical challenges have to date prevented a complete profiling of the levels of *myo*-inositol phosphates (InsPs) and pyrophosphates (PP-InsPs) in mammalian tissues. Here, we have deployed capillary electrophoresis mass spectrometry to identify and record the levels of InsPs and PP-InsPs in several tissues obtained from wild type mice and a newly created PPIP5K2 knockout strain. We observe that the mouse colon harbours unusually high levels of InsPs and PP-InsPs. Additionally, the PP-InsP profile is considerably more complex than previously reported for animal cells: using chemically synthesized internal stable isotope references and high-resolution mass spectra, we characterize two new PP-InsP isomers as 4/6-PP-InsP_5_ and 2-PP-InsP_5_. The latter has not previously been described in nature. The analysis of feces and the commercial mouse diet suggests that the latter is one potential source of noncanonical isomers in the colon. However, we also identify both molecules in the heart, indicating unknown synthesis pathways in mammals. We also demonstrate that the CE-MS method is sensitive enough to measure PP-InsPs from patient samples such as colon biopsies and peripheral blood mononuclear cells (PBMCs). Strikingly, PBMCs also contain 4/6-PP-InsP_5_ and 2-PP-InsP_5_. In summary, our study substantially expands PP-InsP biology in mammals.

## Abstract 2

Capillary electrophoresis mass spectrometry demonstrates that the inositol pyrophosphates 2-PP-InsP5 and 4- or 6-PP-InsP5 exist in different mammalian tissues in addition to canonical isomers.

## Graphical abstract

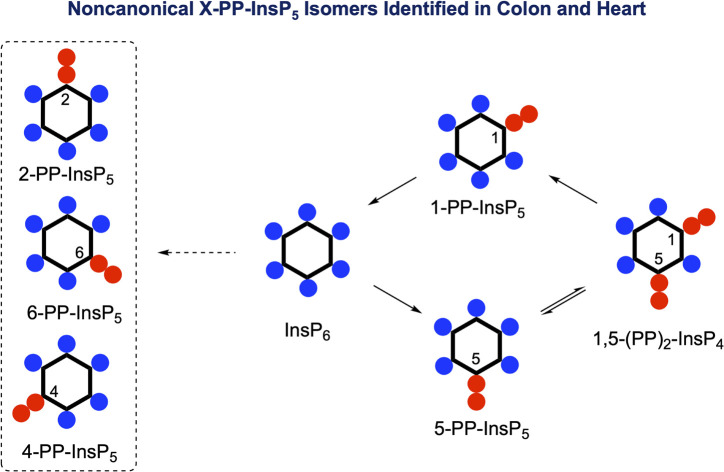

Inositol phosphates (InsPs) and pyrophosphates (PP-InsPs) are a complex signalling hub with diverse functions in eukaryotes.^1–3^ PP-InsPs have specialized physicochemical properties and biological functions that attract widespread interest.^4–7^ They occur as distinct isomers of differentially phosphorylated metabolites of InsP_6_ (phytic acid and phytate). The current literature suggests that in yeast and mammals these phosphorylation reactions occur selectively and successively in the 5- and 1-positions (Fig. 1) leading to 5-PP-InsP_5_ and 1,5-(PP)_2_-InsP_4_, respectively.^8,9^ In plants and slime-mold, 4/6-PP-InsP_5_ has been identified as the main isomer, with the absolute configuration of the biologically relevant enantiomer remaining unknown.^10,11^

**Fig. 1:**
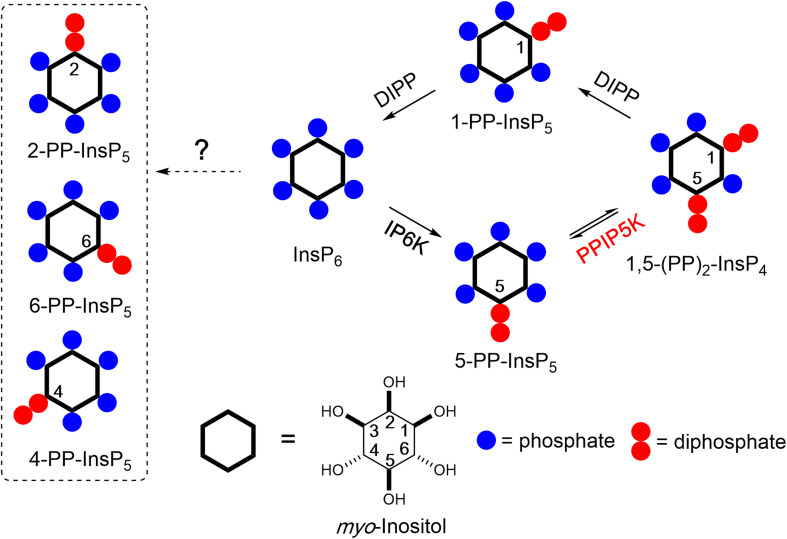
Main metabolic reactions that determine the turnover of inositol pyrophosphates in mammalian cells. Three isoforms of IP6Ks phosphorylate the 5-position of InsP_6_ and two isoforms of PPIP5Ks phosphorylate the 1-position with a preference for 5-PP-InsP_5_ over InsP_6_. The question mark indicates that an unknown pathway is responsible for the synthesis of the 4/6-PP-InsP_5_ and 2-PP-InsP_5_ identified in the current study (inside dotted box, note that 4/6-PP-InsP_5_ are enantiomers). PPIP5Ks also harbour a phosphatase domain catalyzing dephosphorylation 1,5-(PP)_2_-InsP_4_. DIPPs are specialized phosphatases that degrade the phosphoric anhydrides in PP-InsPs.

Kinases and phosphatases that synthesize and metabolize PP-IPs are distributed throughout all eukaryotic kingdoms.^7,12^ In mammals, there are three isoforms of IP6Ks that add a β-phosphate at position 5 and two isoforms of PPIP5Ks that add a β-phosphate at position 1.^8^

Most of the research into PP-InsP turnover in mammalian cells has relied on separation by HPLC of extracts of ^3^H-inositol radiolabeled cells, although in more recent years a more generally accessible PAGE technique has proved useful.^13,14^ This body of work has consistently concluded that 5-PP-InsP_5_ is the most abundant PP-InsP isomer (generally <10% of InsP_6_ levels). The levels of 1,5-(PP)_2_-InsP_4_ and 1-PP-InsP_5_ are approximately 10-fold and 50-fold lower, which are below the PAGE detection limit.^14–16^ The relative ease with which 5-PP-InsP_5_ abundance can be measured has in large part driven the field's primary focus on this isomer. For example, this PP-InsP has been reported to regulate insulin signalling, exocytosis, processing body formation, intracellular protein localization, and bioenergetic homeostasis.^17–22^

More recently, 1,5-(PP)_2_-InsP_4_ has emerged as an independently regulated cellular signal. This facet of PP-InsP signalling first arose from kinetic assessments^23^ of the PPIP5K kinase domain that phosphorylates 5-PP-InsP_5_ to 1,5-(PP)_2_-InsP_4_ and the separate phosphatase domain that degrades 1,5-(PP)_2_-InsP_4_ back to 5-PP-InsP_5_ (see Fig. 1). Moreover, the phosphatase activity is inhibited by elevations in the cellular levels of inorganic phosphate (P_i_), thereby enhancing net 1,5-(PP)_2_-InsP_4_ production independently of any changes in 5-InsP_7_ levels.^23,24^ As a consequence, the net kinase and phosphatase activities are tied to cellular energy and phosphate homeostasis.^3,25^ It has since been demonstrated that 1,5-(PP)_2_-InsP_4_ stimulates P_i_ efflux from mammalian cells through an interaction with an SPX domain on the transmembrane XPR1 protein.^26,27^ Moreover, pharmacologic inhibition of IP6Ks in mammals (rodents and monkeys), which restrains PP-InsP_5_ and 1,5-(PP)_2_-InsP_4_ synthesis (see Fig. 1), leads to attenuation of systemic hyperphosphatemia through inactivation of XPR1; these findings are an important milestone for potential pharmacological treatment of chronic kidney disease.^28^ Naturally occurring human variants of PPIP5K2 have been associated with deafness^29^ and keratoconus.^30^ Recently, [^3^H]inositol-radiolabeling of a hematopoietic stem cell line from a PPIP5K2^−/+^ mouse indicated that 1,5-(PP)_2_-[^3^H]InsP_4_ levels are no different from those in a typical culture medium (data for PPIP5K2^−/−^ cells were not reported).^31^ In such circumstances, it has become more important to be able to accurately assay dynamic fluctuations in 1,5-(PP)_2_-InsP_4_ concentrations.

A portfolio of additional methods has been introduced that can assay the mass levels of (PP)-InsPs in extracts of mammalian and plant cells, including using transition metals (*e.g.* Fe and Y) and absorbance detection (metal dye detection, MDD)^32–34^ and the coupling of in-line mass spectrometry to hydrophilic interaction liquid chromatography (HILIC) and metal-free C_18_ reversed phase columns.^28,35,36^ NMR detection with ^13^C enriched inositol is another recent and promising addition to the analytical portfolio.^37^ In 2020, capillary electrophoresis (CE) with mass spectrometry compatible buffers was reported for PP-InsP analytics, with only nanoliter sample consumption and accurate isomer assignment and quantitation by using stable isotope internal reference compounds.^38^

We now significantly expand the value of our new PP-InsP profiling techniques through our identification of substantial cellular quantities of mammalian 4/6-PP-InsP_5_ and 2-PP-InsP_5_ (see Fig. 1) based on comigration with reference compounds and high-resolution mass spectra. This conclusion is facilitated by adapting a recently developed ^18^O phosphate labelling approach^39^ in order to stereoselectively synthesize 4-PP-InsP_5_ to use as a heavy internal standard. Finally, it was our goal to optimize CE-MS to monitor the complete array of PP-InsPs from human patient tissues. For this work, we selected colon biopsies and peripheral blood mononuclear cells including enriched T cell subpopulations (PBMCs, CD8^+^). Strikingly, we also identify 4/6-PP-InsP_5_ and 2-PP-InsP_5_ in PBMCs that are particularly enriched in a CD8^+^ T cell preparation. Overall, this dramatic increase in the complexity of PP-InsP metabolism indicates that their biological significance has been greatly underestimated.

## Results

**Fig. 2:**
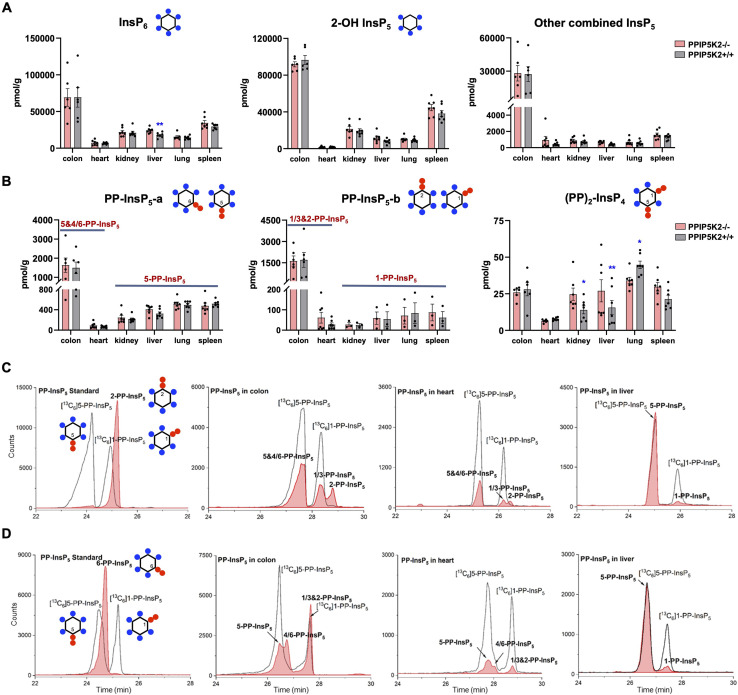
Profiling of PP-InsPs in mouse tissues (wild type *vs.* PPIP5K2 knockout) and observation of new isomers. (A) Profiling of InsP_6_, 2-OH InsP_5_, and the total for all other InsP_5_ isomers (4/6-OH InsP_5_, 1/3-OH InsP_5_, and 5-OH InsP_5_). (B) PP-InsP_5_-a and PP-InsP_5_-b refer to two base-line resolved peaks. The tentative identification of the components of each peak (in bold font) is described in the text. Data for (A) and (B) are indicated as means ± SEM, *n* = 7 and for the colon, *n* = 6. *P < 0.05 and **P < 0.01, Student's *t*-test. (C) Extracted ion electropherograms (EIEs) of [^13^C] labeled internal reference compounds (black lines) plus a 2-PP-InsP_5_ standard (red trace) or PP-InsP_5_ extracts from the mouse colon, heart and liver, resolved with BGE A containing 35 mM ammonium acetate titrated with ammonium hydroxide to pH 9.7. D EIEs of [^13^C]-labeled internal reference compounds (black lines) plus (left panel) a 6-PP-InsP_5_ standard (red trace) or PP-InsP_5_ extracts from the mouse colon, heart and liver (red trace), resolved with BGE B containing 40 mM ammonium acetate titrated with ammonium hydroxide to pH 9.0.

With an established protocol that uses TiO_2_ beads, we extracted and enriched InsPs and PP-InsPs from different mouse tissues.^14,38^ The enriched samples were analyzed by CE-QQQ using the same background electrolyte (35 mM ammonium acetate adjusted to pH 9.7 with NH_4_OH, *i.e.*, BGE-A) that we deployed in our previous study.^38^ Samples were spiked with internal heavy isotope reference compounds (^13^C labels) of several different InsPs and PP-InsPs for assignment and quantitation. This is the first time that this method has been applied to any animal tissue for the quantification of the levels of the least abundant PP-IPs, namely 1,5-(PP)_2_-InsP_4_ and 1-PP-InsP_5_ (for representative examples see Fig. 2C and ESI Fig. 1†).

We also used this method to compare InsP and PP-InsP levels in multiple mouse tissues, including the colon, heart, kidney, liver, lung and spleen (Fig. 2A–C). These molecules were generally least abundant in the heart. It is worth mentioning that other minor InsP_5_ isomers including 4/6-OH InsP_5_, 1/3-OH InsP_5_, and 5-OH InsP_5_ have also been identified and quantified (see representative examples obtained from the mouse colon and mouse heart; ESI Fig. 2†), while 2-OH InsP_5_ was always by far the predominant isomer in all investigated mouse tissues (Fig. 2A).

Compared to other tissues, the colon is notable for containing substantially higher levels of InsP_6_ (2- to 5-fold), 2-OH-InsP_5_ (2- to 10-fold) and the sum of the remaining, quantitatively more minor InsP_5_ isomers (19- to 52-fold). The colon also contains much higher levels of PP-InsP_5_ isomers (Fig. 2A–C). In most of the studied tissues (kidney, liver, lung, and spleen), two baseline-resolved PP-InsP_5_ signals were observed (labeled ‘a’ and ‘b’), which co-eluted precisely with internal standards of [^13^C_6_]5-PP-InsP_5_ and [^13^C_6_]1-PP-InsP_5_, respectively, in each of two different BGE conditions (Fig. 2C and ESI Fig. 3A†). In these tissues, the relative proportion of 1-PP-InsP_5_ to 5-PP-InsP_5_ (approximately 1 to 7) is higher than that determined by our previous CE analysis of a line of immortalized HCT116 cells (1 to 13);^40^ a ratio of only 1 to 50 was previously obtained by HPLC analysis of [^3^H]inositol-labeled extracts of immortalized cells.^41^

An unexpected outcome of the EIE obtained using BGE-A was that the PP-InsP-b signals derived from the colon and heart split into two approximately equally sized peaks that are incompletely resolved; the earlier-eluting peak comigrated with an internal standard of [^13^C_6_]1-PP-InsP_5_ (Fig. 2C). The elution time of the second peak corresponds precisely to the elution time of a replicate sample spiked with an internal standard of 2-PP-InsP_5_ (ESI Fig. 4†). In addition, there is an indication that the PP-InsP-a signal derived from the colon also separates into two incompletely resolved peaks (Fig. 2C). To pursue the latter observation, we reran the samples with the background electrolyte adjusted to 40 mM ammonium acetate titrated with ammonium hydroxide to pH 9.0 (*i.e.*, BGE-B). This procedure extended the peak-to-peak resolution within the PP-InsP-a signal to the extent that its two components are also visible in the extracts prepared from the colon and heart (Fig. 2D). Note that, in contrast, the use of BGE-B did not perturb the coelution of internal standards of [^13^C_6_]5-PP-InsP_5_ and [^13^C_6_]1-PP-InsP_5_ with PP-InsP-a and PP-InsP-b signals, respectively, that were prepared from the kidney, liver, lung and spleen (Fig. 2C and D; ESI Fig. 3A and B†). However, we do not exclude that matrix effects in other tissues would blur the presence of low levels of additional PP-InsP isomers.

In this set of experiments with BGE-B, the first component of PP-InsP-a extracted from the colon comigrates with the internal standard of [^13^C_6_]5-PP-InsP_5_ and the second component of PP-InsP-a has an elution time that matches that of a standard of 6-PP-InsP_5_ from separate runs (Fig. 2D). Thus, we tentatively identify the second component of PP-InsP-a as 4/6-PP-InsP_5_ and by a process of elimination we suggest that the second component of PP-InsP-b is 2-PP-InsP_5_. Moreover, the proposed nature of 1/3-PP-InsP_5_, 2-PP-InsP_5_, 5-PP-InsP_5_ and 4/6-PP-InsP_5_ from the colon is also consistent with their high-resolution mass spectra collected by using a CE-qTOF system (ESI Fig. 5†). Other potential candidates with an identical mass, such as triphosphates of inositol-tetrakisphosphates (*e.g.* 5-PPP-InsP_4_), have been described so far only *in vitro*.^42^ The *myo*-configuration for these new PP-InsPs seems likely, since there is no prior identification of any other multiply phosphorylated inositol stereoisomers in mammals.

It is notable that in the colon we estimate that the levels of 1-PP-InsP_5_ (*i.e.*, half of PP-InsP-b) and 5-PP-InsP_5_ (*i.e.*, half of PP-InsP-a) are approximately equivalent (Fig. 2C and D); this observation implies that we must profoundly modify prior perceptions of 1-PP-InsP_5_ as a quantitatively minor constituent of mammalian cells and/or consider the possibility that the enantiomer 3-PP-InsP_5_ is also present. Currently applied methods do not resolve the enantiomers.

**Fig. 3:**
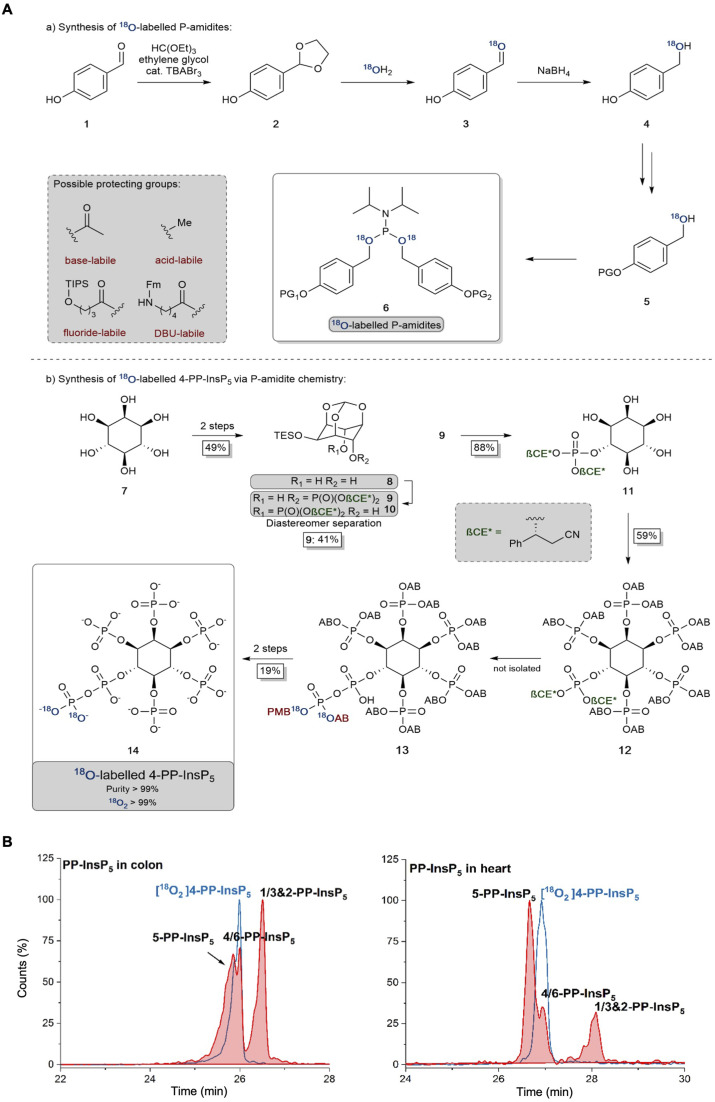
(A) Synthesis and application of ^18^O labeled P-amidites with diverse protecting group patterns and their application to a late-stage labeling 4-PP-InsP_5_ synthesis. AB: acetoxybenzyl and PMB: *para*-methoxybenzyl. (B) Separation of 5-PP-InsP_5_ and 4/6-PP-InsP_5_ (filled red plots) from mouse colon and heart samples using BGE-B and assignment of the isomer with the new internal reference compound [^18^O_2_] 4-PP-InsP_5_ (blue plot) as either 4-PP-InsP_5_ or 6-PP-InsP_5_. EIEs (PP-InsP_5_ and [^18^O_2_] PP-InsP_5_) are scaled to the largest peak indicated as 100%.

The 2-PP-InsP_5_ isomer has not previously been identified in any biological material, possibly because it is both unexpected and only present at relatively low levels. In contrast, 4/6-PP-InsP_5_ was recently discovered to be a major PP-InsP isomer in plants.^10^ Clearly, the latter is also a quantitatively important isomer in the mouse colon and heart (Fig. 2), and so it was particularly important to further validate its nature. Thus, we have developed a synthetic route to the preparation of enantiomerically pure [^18^O_2_]4-PP-InsP_5_ to deploy as an internal standard for additional chromatographic resolutions (see Fig. 3).

We have also recorded 1,5-(PP)_2_-InsP_4_ levels in mouse tissues (Fig. 2B). These varied over a 5-fold range, with the lowest levels in the heart and the highest in the lung; as far as we are aware, no previous study has provided such data. This accomplishment enabled us to determine the impact upon 1,5-(PP)_2_-InsP_4_ levels in a newly created PPIP5K2 knockout mouse (ESI Fig. 6 and 7†). No abnormal phenotype in the KO was observed. The litter size and gender distribution were not modified. No gross anatomical phenotype was observed during tissue collection. Food intake or energy expenditure (metabolic rate and physical activity) were unchanged (ESI Fig. 8†). We did not conduct any behavioral phenotyping.

The knockout only resulted in a statistically significant reduction in 1,5-(PP)_2_-InsP_4_ levels in the lung tissue (Fig. 2B). In fact, 1,5-(PP)_2_-InsP_4_ levels trended higher in several PPIP5K2 knockout tissues compared to the wild-type, and in the kidney and liver this effect was statistically significant. Although this might initially seem a counter-intuitive outcome, it is possible that in these two tissues the loss of the PPIP5K2 1,5-(PP)_2_-InsP_4_-phosphatase domain may have a larger metabolic effect than the loss of the 5-PP-InsP_5_ kinase domain. The knockout did not elicit a statistically significant impact on 1,5-(PP)_2_-InsP_4_ levels in either the colon or the heart. The observation of tissue dependent variability in PP-InsP signaling brought about by PPIP5K2 knockout may depend in part on the extent to which PPIP5K1 compensates for the deletion of PPIP5K2 catalytic activity, although no such effect was evident in the liver (ESI Fig. 6 and 7†). Note also that the PPIP5K2 KO did not have off-target effects on any of the other InsPs and PP-InsPs analyzed in this study (Fig. 2A and B), except that InsP_6_ was increased in the PPIP5K KO liver.

We could not derive sufficient purified amounts of the putative 4/6-PP-InsP_5_ for NMR analysis to further corroborate the identity of this isomer. So instead, we generated a reference compound with a heavy isotope label to serve as an internal standard for CE-MS. We reasoned that the comigration of this compound under different separation conditions would serve as a strong indication that it is indeed 4/6-PP-InsP_5_ in its *myo*-configuration. The enzymes for plant 4/6-PP-InsP_5_ synthesis are not yet known and so an enzymatic synthesis starting from InsP_6_ of the reference compound with ^13^C labels was not possible.^43^ A fully chemical synthesis from expensive ^13^C glucose in a multi-step linear approach was deemed not feasible.^37^ We thus relied on our recently developed ^18^O phosphate labeling approach in which the expensive isotopic label can be introduced in the penultimate step of the synthesis.^39^

In brief, ^18^O labeled phosphoramidites (P-amidites) with high ^18^O/^16^O ratios are key to the synthesis. These high ratios can be obtained by the strategy shown in Fig. 3A(a). *Para*-hydroxybenzaldehyde is transformed into its acetal 2, which is then hydrolyzed in the presence of 99% ^18^O enriched water. The aldehyde 3 is directly reduced to stable alcohol 4, which can then be protected on the phenol with diverse protecting groups (in the case described here simply acetate giving the acetoxybenzyl (AB) protecting group). The alcohols 5 are then transformed into P-amidites of the general structure 6, enabling diverse protecting group patterns and high ^18^O enrichment. The inositol structure is assembled as reported previously,^44–47^ as shown in Fig. 3A(b). While strictly a desymmetrization was not required and the generation of racemic 4/6-PP-InsP_5_ would have been sufficient, we still generated the enantiomerically pure compound for potential future applications. Desymmetrization was achieved from intermediate protected diol 8, which was reacted with an unsymmetric P-amidite containing chiral protecting groups (β-CE*, an arylated enantiomerically pure variant of the β-cyanoethyl protecting group). The obtained diastereomeric mixture was separated and then the inositol protecting groups were removed giving pentaol 11. 11 was phosphorylated to protected InsP_6_12 with orthogonal protecting groups (β-CE*) in the 4-position.^44^ Selective deprotection in that position then enables the introduction of the ^18^O labeled phosphate bearing two ^18^O oxygen atoms (M + 4). Global deprotection gave [^18^O]_2_ 4-PP-InsP_5_14 in 99% purity with >99% isotopic enrichment as determined by CE-MS. This reference compound was then dissolved in water and its concentration was determined by quantitative ^1^H- and ^31^P-NMR.

Fig. 3B demonstrates the first application of this newly generated isotopologue. Briefly, both colon and heart samples were spiked with the new reference and we utilized the optimized BGE-B that is capable of 5-PP-InsP_5_ and 4/6-PP-InsP_5_ separation. Masses were recorded and an identical migration of the unknown analyte with our reference in the same matrix was found, strongly suggesting that it is indeed 4/6-PP-InsP_5_ that has been measured for the first time in mammalian tissues.

**Fig. 4:**
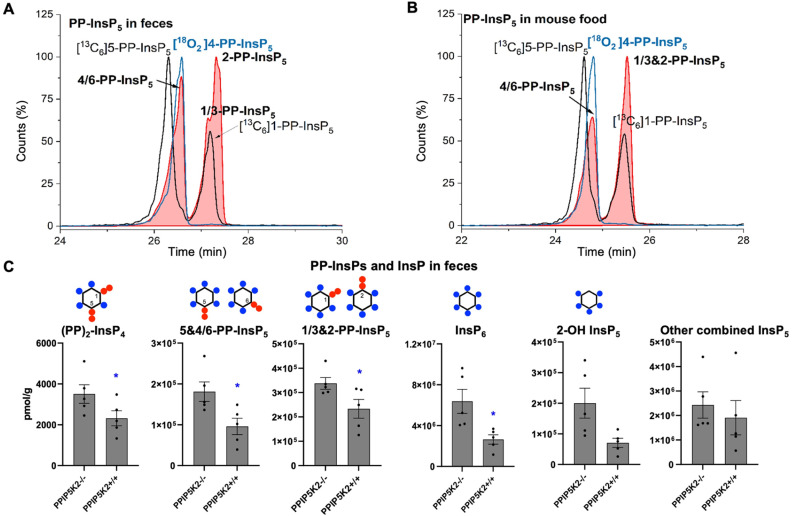
(A) CE-MS analysis of PP-InsP_5_ in mouse feces (filled red plots) with internal references of [^13^C_6_]5-PP-InsP_5_ and [^13^C_6_]1-PP-InsP_5_ (black plot) and [^18^O_2_]4-PP-InsP_5_ (blue plot) using BGE B. 4/6-PP-InsP_5_ isomer is identified in mouse feces as well. (B) Analysis of PP-InsP_5_ in mouse food the same as in (A), which contains high levels of this 4/6-PP-InsP_5_ isomer. (C) Profiling of PP-InsPs and InsPs in mouse feces (wild type *vs.* PPIP5K2 knockout). Data are indicated as means ± SEM, *n* = 5. *P < 0.05, and Student's *t*-test.

To understand the complexity of the profiles of InsPs and PP-InsPs in the colon in an organismal context, we additionally analyzed mouse feces and found them to contain very high levels of most analytes (Fig. 4A and C, ESI Fig. 9†). Moreover, neither PP-InsP peak co-eluted precisely with internal standards of either 5-PP-InsP_5_ or 1-PP-InsP_5_ again pointing towards the existence of both 4/6-PP-InsP_5_ and 2-PP-InsP_5_. In fact, the two new isomers are the most abundant analytes we detect (Fig. 4A). Interestingly, the PPIP5K knockout contained increased levels of all analytes in feces. We excluded that this was due to differences in food intake (ESI Fig. 8†). It may be possible that such changes are caused by different expression of digestive enzymes of PP-InsPs.

We next investigated if the mouse laboratory diet might contribute to the unprecedented complexity of the colonic PP-InsP profile. We provided mice with the “Rodent NIH-31 Open Formula Autoclavable Diet”, much of which is of plant origin. This is significant because recent work has determined that the quantitatively most important PP-InsP isomer in plants is one that had previously been overlooked, namely 4/6-PP-InsP_5._^10^ Indeed, our internal standards allowed us to conclude that large amounts of 4/6-PP-InsP_5_ were present in the mouse diet, although a precise quantification was hindered by insufficient separation of the 4/6- and 5-PP-InsP_5_ peaks from the PP-InsP_5_-a peak (Fig. 4B). Nevertheless, the latter was smaller than the PP-InsP_5_-b peak, which likely comprises a mixture of 1-PP-InsP_5_ and 2-PP-InsP_5_.

Our results present the possibility that the diet might be the source of the colon's unusually high levels of InsP_6_ and PP-InsPs, as well as the more complex PP-InsP profile. Furthermore, 2-OH InsP_5_ is the minor InsP_5_ isomer in mouse feces and also in mouse food (ESI Fig. 10†), in contrast to it being the major InsP_5_ in the colon. This result suggests that the exceptional PP-InsPs and InsP_6_ profile in the colon are not due to contamination from feces during sample preparation. In this case, endocytosis of dietary InsP_6_ and PP-InsPs by colonic epithelial cells should be considered as a viable possibility.

**Fig. 5:**
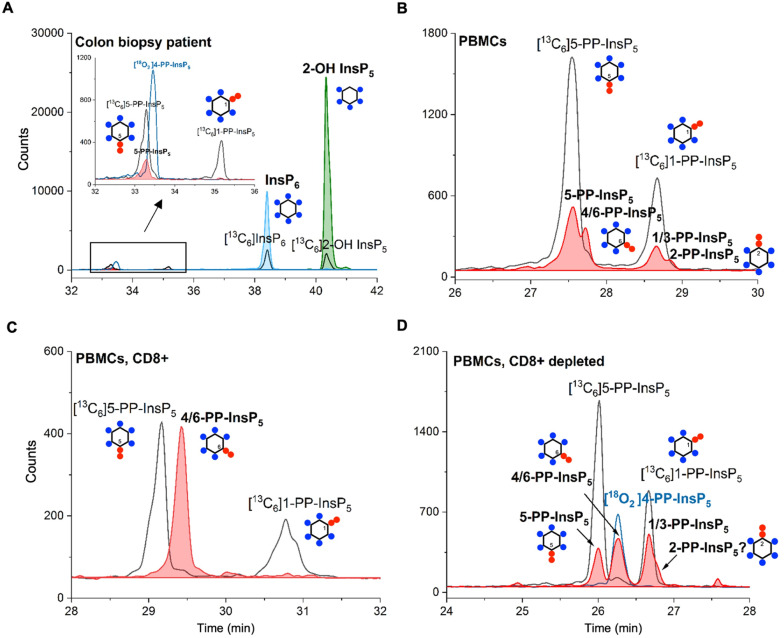
(A) CE-MS analysis of a human colon tissue biopsy (18 mg) enables the identification of several important inositol phosphate (InsP_6_ and InsP_5_) and pyrophosphate (5-PP-InsP_5_) isomers. (B) 4/6-PP-InsP_5_ is identified in PBMCs by CE-MS analysis. The electropherograms are representative of independent biological triplicates giving comparable results. (C) 4/6-PP-InsP_5_ is enriched in a CD8^+^ T-cell preparation and is also present in the CD8^+^ depleted PBMC pool (D). It is assigned by its exact same migration time as [^18^O_2_] 4-PP-InsP_5_. PP-InsP_5_ (filled red plot) isomer identification is achieved with the aid of [^13^C_6_]5-PP-InsP_5_, [^13^C_6_]1-PP-InsP_5_ (black plot) and [^18^O_2_] 4-PP-InsP_5_ (blue plot).

Finally, in order to demonstrate the sensitivity of the method and its potential in translational research, we obtained human samples for enrichment and profiling. We analyzed one 18 mg wet tissue colon biopsy, which was sufficient to profile the main PP-InsP and InsP contents (Fig. 5A). Only canonical isomers were identified, *i.e.* 5-PP-InsP_5_, InsP_6_, and 2-OH InsP_5_. We additionally analyzed peripheral blood mononuclear cells (PBMCs; Fig. 5B) from donors, and also CD8^+^ T-cells enriched from the PBMC pool by FACS (see the ESI†). Strikingly, in one such enriched sample, we identified 4/6-PP-InsP_5_ as the sole PP-InsP isomer (Fig. 5C). Of note, the CD8^+^ depleted PBMC pool (Fig. 5D) also contained 4/6-PP-InsP_5_ as well as 5-PP-InsP_5_ and the latter was identified as the minor isomer. Moreover, a peak comigrating with 2-PP-InsP_5_ was identified in PBMCs (Fig. 5B) and can be tentatively assigned to a shoulder of the peak of the CD8^+^ depleted fraction (Fig. 5D). CD8^+^ enrichment did not provide enough material for analysis in all samples studied, so it remains unclear whether the surprising 4/6-PP-InsP_5_ enrichment is generally found in CD8^+^ cells from different donors. However, our analysis now firmly establishes that this new isomer is of mammalian origin.

## Conclusions

We have applied CE-MS profiling to delineate a more sophisticated picture of InsP and PP-InsP distributions in metazoan samples. Thus, inositol pyrophosphate signalling appears even more complex than previously thought. The CE-MS method also has sufficient sensitivity to profile for the first time biopsies from human patients and PBMCs including isolated CD8^+^ T-cells from human blood. We obtain several unexpected results based on the high separation efficiency of capillary electrophoresis that have gone undetected with recently developed LC-MS approaches.^28,35,36^ In particular, we identify very high levels of PP-InsPs in colon tissue, which are potentially endocytosed from the laboratory diet, including large quantities of the putative noncanonical 4/6 and 2-PP-InsP_5_ isomers. Our data therefore represent a paradigm shift in our understanding of dietary influences upon PP-InsP metabolism and signaling in the colon. While 4/6-PP-InsP_5_ and 2-PP-InsP_5_ in the colon could possibly originate from the endocytosis of food constituents, this phenomenon cannot apply to heart samples as well as human PBMCs. Consequently, it appears that 4/6-PP-InsP_5_ and 2-PP-InsP_5_ can also be synthesized by mammals.

Our new isomer assignments are based on the exact mass determination and exact comigration with standards of both PP-InsPs, including a novel synthetic 4-PP-InsP_5_ bearing two ^18^O oxygen isotope labels. Future studies must now address the enantiomeric identity of the new metazoan 4/6-PP-InsP_5_ as well as a complete structural assignment of 2-PP-InsP_5_ by NMR to firmly establish *myo*-configuration and exclude other potential constitutional isomers of the same mass and identical migration during CE. The colonic uptake, dynamic regulation, unknown enzymology and functions of these new isomers will be productive directions for future research, including their presence in the central nervous system. With the ability to profile PP-InsPs from human biopsies and blood samples, their establishment as potential disease biomarkers will also become an important future endeavor.

## Data availability

Additional data can be found in the ESI.†

## Author contributions

DQ and CG designed, performed, and evaluated analytical experiments. CG was responsible for the animal experiments. SBS supervised animal experiments. GL and VE performed biopsy extractions and analytics. KR conducted the chemical synthesis. TB and LS isolated and extracted PBMCs. BB supervised human tissue extractions and designed experiments. HJJ and SBS conceived the idea of the project and designed experiments. HJJ, DQ, CG, and SBS wrote the manuscript. All authors provided feedback on experimental design and contributed to manuscript revisions.

## Conflicts of interest

There are no conflicts to declare.

## Supplementary Material

SC-014-D2SC05147H-s001
